# Biomechanical Behavior of Dynamic vs. Static Distal Locking Intramedullary Nails in Subtrochanteric Femur Fractures

**DOI:** 10.3390/bioengineering10101179

**Published:** 2023-10-11

**Authors:** Carmen Martínez-Aznar, Jesús Mateo, Elena Ibarz, Luis Gracia, Jorge Rosell, Sergio Puértolas

**Affiliations:** 1 Department of Orthopaedic Surgery and Traumatology, Reina Sofía Hospital, 31500 Tudela, Spain; 2 Department of Orthopaedic Surgery and Traumatology, Miguel Servet University Hospital, 50009 Zaragoza, Spain; 3 Department of Mechanical Engineering, University of Zaragoza, 50018 Zaragoza, Spain; 4 Aragón Institute for Engineering Research, 50018 Zaragoza, Spain

**Keywords:** intramedullary nail, subtrochanteric fracture, osteosynthesis, finite element analysis, biomechanics

## Abstract

Objective: Hip fractures are one of the most frequent fractures presenting to the emergency department and orthopedic trauma teams. The aim of this study was to determine the best indication and therapeutic technique for subtrochanteric fractures and unifying criteria when choosing the most suitable type of nail. Materials and methods: To analyze the influence of the material and the type of distal locking of intramedullary nails (static or dynamic), a femur model with a fracture in the subtrochanteric region stabilized with a long Gamma intramedullary nail was applied using finite element method (FEM) simulation. Results: The mechanical study shows that titanium nails allow for greater micromobility at the fracture site, which could act as a stimulus for the formation of callus and consolidation of the fracture. In the mechanical study, the type of distal locking mainly affects mobility at the fracture site and stress in the cortical bone around the distal screws, without in any case exceeding values that may compromise the viability of the assembly or that may result in detrimental effects (in terms of mobility at the fracture site) for the consolidation process. Conclusion: Subtrochanteric fractures treated with titanium nail and static distal locking is safe and does not hinder consolidation.

## 1. Introduction

Hip fractures are one of the most common problems encountered by orthopedic surgeons. In Spain, the incidence of hip fractures is 60,000 cases per year, occurring at an average age of 82 years. The prevalence of this type of fracture is increasing, and it is expected that by 2050, there will be more than 6 million cases worldwide [1,2].

With proximal femur fractures, subtrochanteric fractures are the least frequent and generally also the most complex to treat. There are various treatment options for subtrochanteric fracture of the femur: extramedullary devices (sliding screw plates or fixed angled plates) [3,4] and intramedullary devices. Both intramedullary nailing and extramedullary plating have been effective treatment options for subtrochanteric fractures. Xie H. et al. [5], in their meta-analysis, compared these two surgical techniques, and they found that intramedullary fixation is associated with significantly shorter operation time, less intraoperative blood loss, shorter length of incision and length of stay, as well as a lower rate of fixation failure, reoperation, and a higher rate of excellent and good functional results than extramedullary fixation for subtrochanteric fractures. In addition, proximal femoral locking plates are associated with a high complication rate [6].

Currently, there is widespread acceptance that treatment with intramedullary devices (nails) is the most effective, both for its biomechanical advantages and for its lower incidence of postoperative complications [7].

Since the introduction of cephalomedullary nails, there has been an ongoing discussion regarding the most appropriate nail configuration. Currently, there is no clear criterion regarding the material to be used (stainless steel or titanium), nor regarding the use and placement of locking screws for the treatment of subtrochanteric fractures [8,9].

In recent years, bioengineering has applied finite element method (FEM) simulation models to different clinical problems, as they are very useful in the study of the locomotor system [10]. FEM simulation reveals the biomechanical changes induced by the placement of an implant, thereby helping to explain the response of the bone to these changes in the mechanical conditions of transmission of physiological loads [11]. In addition, FEM simulation can modify the characteristics of implants, which allows assessment of the best system and optimization of its behavior [12,13]. As they do not require intervention on a living being, simulation models allow the study to be repeated as many times as necessary, with variations in its conditions.

Despite the advantages of these computational techniques, there are still limited publications relating to studies of the biomechanical behaviors of intramedullary nails in subtrochanteric fractures in the femur. Most of them focus on intramedullary nailing of femoral shaft fractures [14,15,16,17], and very few articles specifically address subtrochanteric fractures [18,19,20,21]. Kwak DK et al. [18], Jang CY et al. [19], and Lee WC et al. [20] presented different works related to the study of the application of short cephalomedullary nails for the treatment of subtrochanteric and intertrochanteric fractures using finite element models. Kwak DK et al. [18] and Jang CY et al. [19] investigated the stresses around the nails and cortical bones to determine the most appropriate type of short cephalomedullary nail for different fracture levels. Lee WC et al. [20] aimed to determine the appropriate length of distal extension of a short proximal femoral nail antirotation (PFNA-II) device to stabilize a specific multifragmentary intertrochanteric femur fracture.

The choice of one material versus another for the composition of the nail is not straightforward, and there is a great diversity of opinions in this regard [22]. Mechanical study by the simulation of models with osteosynthesis of steel and titanium aims to assess the differences between the two materials.

Similarly, there is no consensus in terms of the choice of the type of distal nail locking [23]. Several clinical studies comparing the outcome of dynamic versus static intramedullary nailing in peritrochanteric and shaft fractures of the femur have shown variable results [24,25,26,27,28]. A priori, the main indication for choosing one type of locking versus another lies in the degree of comminution and instability of the fracture, with preferential use in the usual clinical practice of static locking when the fracture is considered relatively unstable.

To date, there have been very few publications on the mechanical outcomes of distal intramedullary locking in subtrochanteric fractures with long cephalomedullary nails. Recently, just a study has been presented regarding the biomechanical performance of short and long cephalomedullary nails with varying numbers of distal screws to stabilize different levels of subtrochanteric fractures [21].

**Figure 1:**
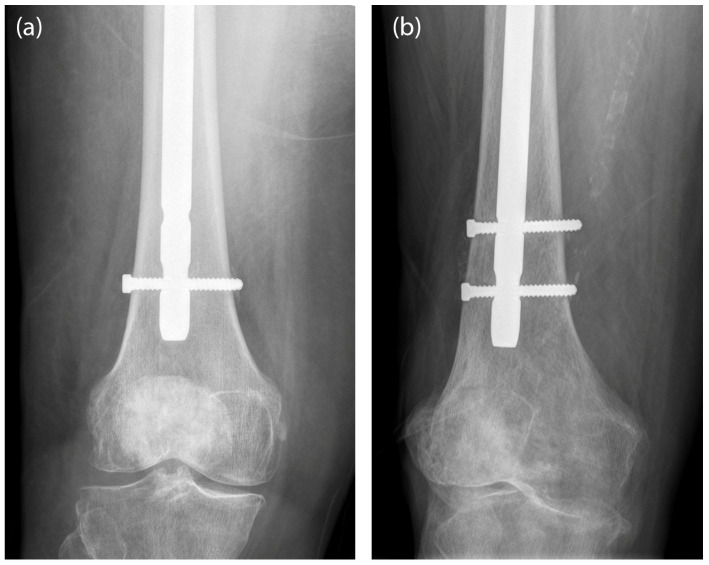
(**a**) Dynamic locking; (**b**) static locking.

The main objective of this study was to analyze the influence of the type of distal locking of intramedullary nails (static or dynamic) on subtrochanteric femur fractures (Figure 1) and which of the two most widely used materials for the manufacture of this type of nails (stainless steel or titanium) provides better results, with the purpose of determining the best indication and therapeutic technique for intramedullary nailing of subtrochanteric fractures, with regard to the selection of the distal interlocking type and the material the nail is made of.

For this purpose, a femur model with a fracture in the subtrochanteric region stabilized with a long Gamma intramedullary nail was applied using FEM simulation. The behavior of osteosynthesis (mobility at the fracture site and stresses generated in osteosynthesis and in the bone), as well as the general stability of the assembly, were assessed with this model by modifying the options of distal locking and the type of nail material.

## 2. Materials and Methods

### 2.1. Model of the Femur and Implants

For the biomechanical study, a three-dimensional (3D) finite element method was applied to a healthy femur. The geometric model of the femur was developed by scanning an accurate replica of the femur of a 55-year-old male donor using a Rolan PICZA LPX-250 3D (Irvine, CA, USA) laser scanner. The scanning was carried out using a rotary scan with a resolution of 0.2 mm, resulting in a point cloud.

**Figure 2:**
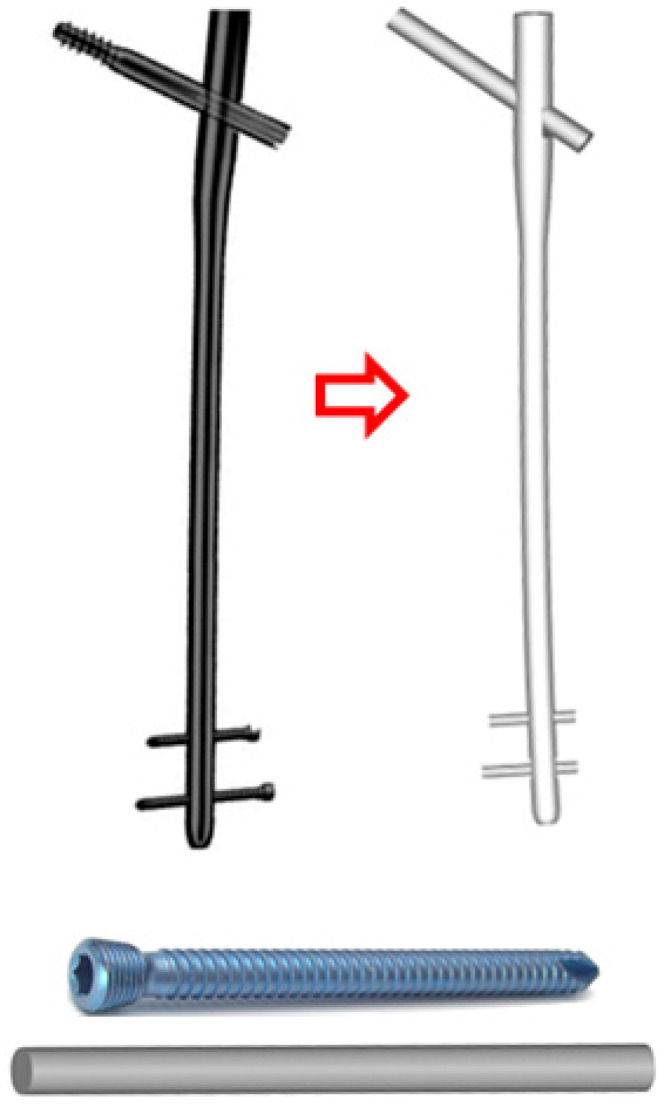
Three-dimensional design of the nail and screws. Actual manufacturer’s design and geometric model for simulations.

The Stryker Long Gamma intramedullary nail (Stryker, Mahwah, NJ, USA) was used in this study. This nail features multiple length options ranging from 280 mm to 460 mm in increments of 20 mm. For the present study, a 360 mm nail was chosen, thus avoiding extreme values and in accordance with the anatomical model obtained. The cervico-diaphyseal angle of the nail studied was 125°, and its diameter was 11 mm, except in the proximal region, which was 15.5 mm in diameter. The nail and the locking screws were assessed using three-dimensional design software, specifically with the design and structural analysis software NX- I-Deas^®^11 [29] (Figure 2).

**Figure 3:**
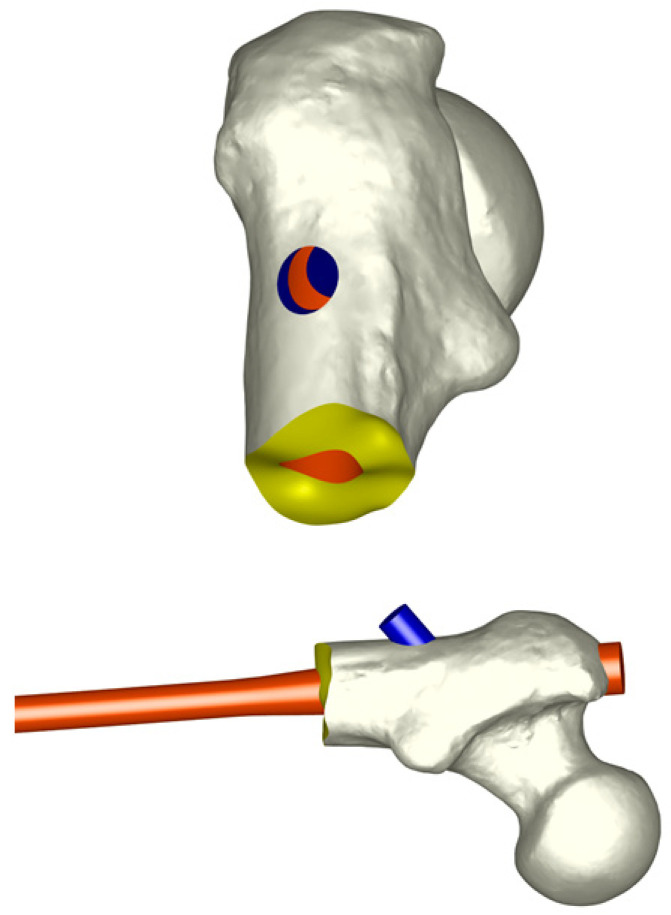
Complete finite element model.

The process of creating the femur model with the nail was also performed using the NX I-DEAS program. For this, a ‘virtual surgery’ was performed in a virtual way, inserting the nail into the femur with the corresponding screws. Once the final geometric model had been prepared, a meshing process was carried out on each of the parts that make up the model (femur, intramedullary nail, and screws) to obtain the complete finite element model (Figure 3). A sensitivity analysis was previously performed to determine the minimal mesh size required for an accurate and precise simulation. For this purpose, a mesh refinement was executed in order to achieve a convergence toward a minimum of the potential energy, both for the whole model and for each of its components, with a tolerance of 1% between consecutive meshes. The final elements used to mesh the different parts of the model were linear tetrahedral elements with an approximate size of 1.5 mm.

**Figure 4:**
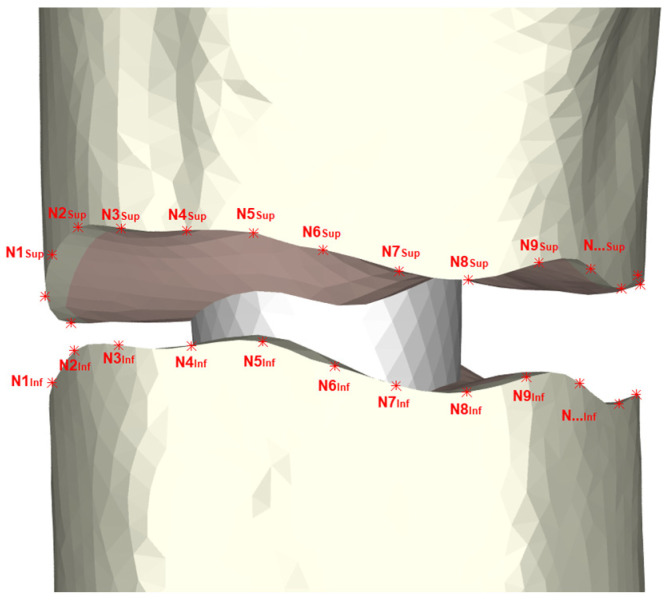
Definition of homologous nodes in the upper and lower regions of the fracture to measure interfragmentary movements.

Relative displacements between the proximal and distal fracture fragments needed to be measured in order to assess the movement at the fracture site. For this purpose, pairs of counterpart nodes were identified by a number at each of the fracture fragments. Movements of each pair of nodes were obtained, which made it possible to assess the relative movements at the fracture site (Figure 4).

### 2.2. Material Properties

Bone tissue is considered a linear and isotropic elastic material. The mechanical properties were assigned to the model using the Abaqus 6.14 program [30] (Table 1).

### 2.3. Contact Modeling

Each part of the osteosynthesis model was meshed independently; therefore, it was necessary to define the contact interactions between the different elements. To model the self-locking screws that anchor perfectly into the cortical bone, a “Tie” type interaction was selected for the cortical bone and locking screw contact zones, simulating the rigid union the screw thread provides. For areas where two or more meshes could potentially come into contact during load application, a “Surface-to-Surface” interaction was defined. A friction coefficient of 0.15 was utilized to specify the contact interaction in accordance with the literature [16,17]. This applied to the contact zones between the screws and trabecular bone, the external surface nail, and the trabecular bone of the medullary canal, as well as the upper and lower fracture surfaces.

### 2.4. Loads and Boundary Conditions

**Figure 5:**
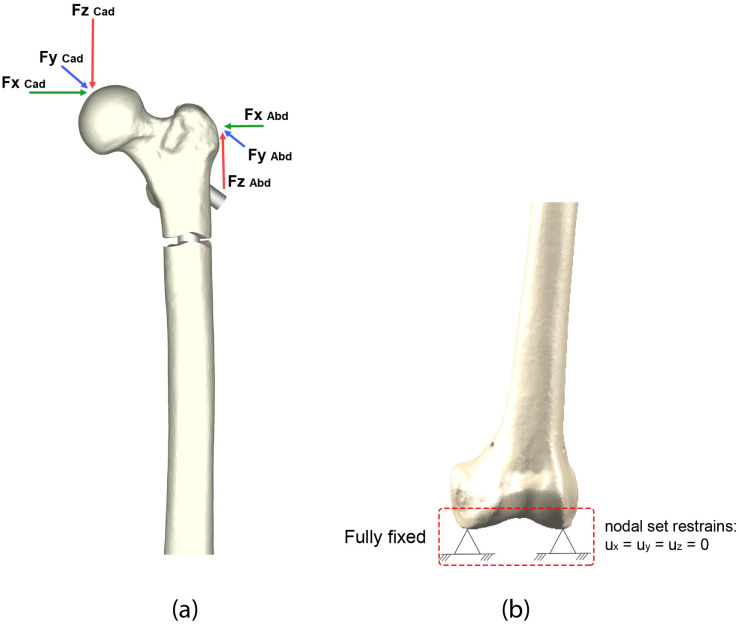
Boundary conditions: (**a**) loads; (**b**) displacement restrains.

In addition to the mechanical properties of the bone, it was necessary to take into account the physiological forces acting on the hip during movement [31]. Therefore, it was necessary to consider the force due to the weight supported by the hip, as well as the forces generated by the muscle groups (Figure 5a). The condylar zone was fully constrained in the distal region, preventing nodal displacements in all directions (Figure 5b). As a consequence, all movements were relative to the immobilized zone.

The OrthoLoad database [32] provides values based on weight; in this case, a 70 kg patient type was chosen. In the study, an incidence of accidental foot support on the ground was simulated in an early postoperative stage so that a load of 25% of the physiological load could be assessed. The loads applied to the hip, according to OrthoLoad, were the reaction and abduction forces of the hip, referred to as 45% of the gait, corresponding to the maximum and most representative load.

Thus, through the development of a computational model based on the finite element method, the biomechanical behavior of the various osteosynthesis models (Table 2), manufactured out of steel and titanium, was studied on a fracture in the femoral subtrochanteric region with a gap of 6 mm with long cephalomedullary nails with dynamic (one screw) and static (two screws) distal locking.

The different computational simulations were made using Abaqus 6.14 software [30].

## 3. Results

### 3.1. Type of Locking

Following are the data from the mechanical study, representing the effects of different locking in osteosynthesis (Table 3). For this purpose, a subtrochanteric inverse fracture with a gap of 6 mm synthesized with a long steel Gamma nail was modeled.

The maximum displacement in the femoral head (maximum overall displacement) underwent subtle changes depending on the distal nail locking. Dynamic osteosynthesis had 3.36% more mobility than static osteosynthesis.

The difference in mobility at the fracture site was greater than the difference in mobility in the femoral head, with increases in mobility at the fracture site of 11.46% in dynamic osteosynthesis with respect to static osteosynthesis.

**Figure 6:**
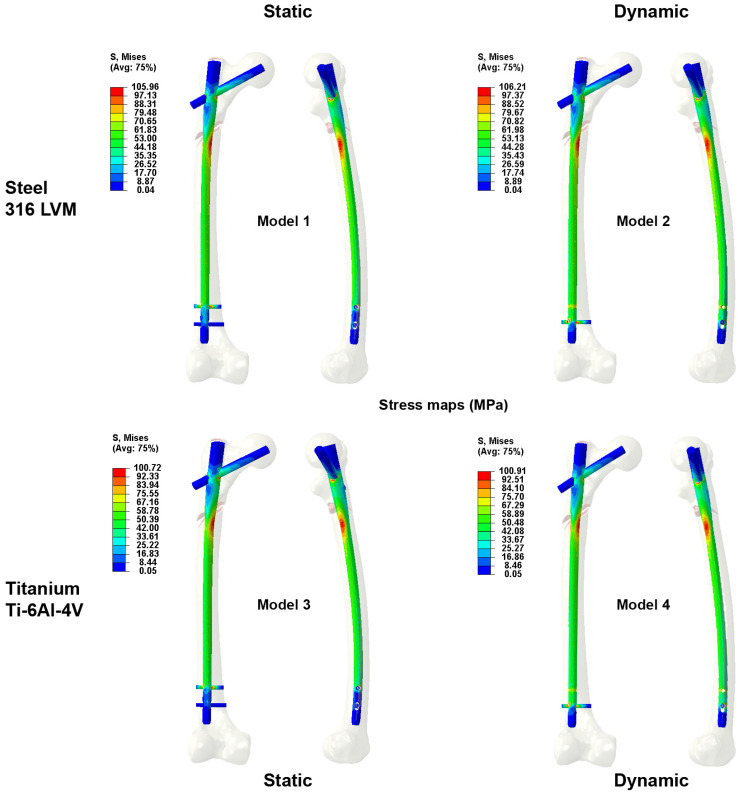
Map of the maximum Von Mises stress values measured at the intramedullary nailing for the osteosyntheses analyzed.

Regarding the stresses in the nail (Figure 6), there was not a significant difference in the stress experienced by the nail depending on the distal locking. The difference between the two did not exceed 1% of the stress. In all cases, the stress levels that appeared in the nail due to the use of 25% of the physiological load were very low compared with the yield limits (Table 1) of both materials.

**Figure 7:**
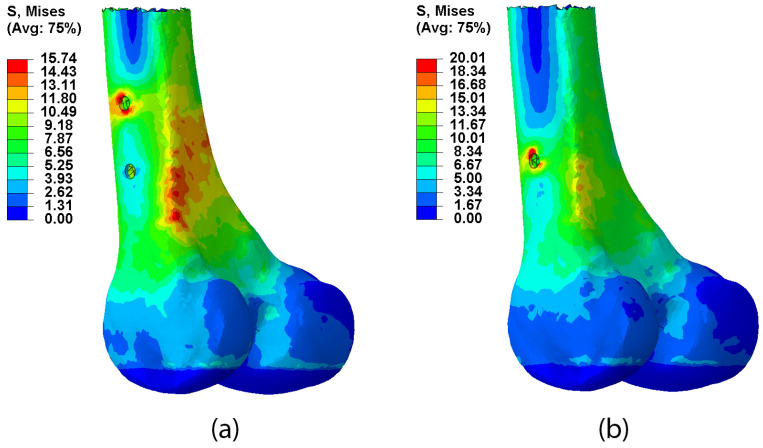
Von Mises stresses for the implanted models in distal region of the femur: (**a**) static locking; (**b**) dynamic locking.

To evaluate the stress supported by the cortical bone, the distal area surrounding the distal screws was chosen to further examine the difference between the placement of one or two screws in this area. In this case, there was a notable difference in the stress on the distal screws depending on their configuration. Dynamic steel osteosynthesis resulted in 27.16% more stress in the cortical bone of the distal femur than the stress found when there were two screws. Moreover, with dynamic locking, the maximum stress occurred in the insertion area of the dynamic distal screw in the cortical bone. However, when using static locking, in addition to reducing the maximum stress, it occurred in the supracondylar area instead of at the insertion point (Figure 7).

The clinical effect of nail dynamization consists of the approximation of the fracture fragments following the axis determined by the intramedullary nail. All physiological loads from the hip are transmitted to the nail through the contact surfaces between the nail and the femoral canal. The greater the interface nail–bone contact, the better the transmission of loads, which leads to less relative mobility of the bone fragments at the fracture site and less stress supported by the nail. In dynamic locking, the load began to be transmitted when the path of the screw in the oval hole came to an end, promoting free mobility between the fragments until the path of the screw came to an end or until the fracture gap was filled. Therefore, we compared the performance of two distal locking screws with the performance of one distal screw where the gap fragment was filled.

### 3.2. Type of Material

Two models were devised for the study of the type of nail material. An inverse subtrochanteric fracture with a 6 mm gap and dynamic distal lock synthesized with a steel nail or titanium nail was performed. The data obtained are shown in Table 4.

In terms of mobility, titanium osteosynthesis allowed for greater mobility both overall and at the level of the fracture site. In our study, the overall mobility (maximum proximal fragment displacement) obtained was 3.97 mm with the steel nail and 5.32 mm with the titanium nail, amounting to a difference of 34.4%. Similarly, the average mobility at the fracture site was 326 µm for the steel nail and 520 µm for the titanium nail, which amounted to a mobility difference of 56.7%.

Regarding the stresses experienced by the femur, the cortical bone has three mechanically significant stress zones: the contact zone with the cephalic screw, the contact zone with the distal screw, and the diaphyseal medullary canal. In this case, the first was chosen to assess the stresses at the level of the cortical bone since, clinically, it has been observed that the Z-effect—which describes the migration of the cephalic screw through the femoral head [33]—is a potential cause of failure of intramedullary osteosynthesis.

Unlike what happens when stress values in the nail were measured, the cortical bone at the proximal level underwent higher rates of stress when the nail used was made of titanium. The titanium nail resulted in stresses in the cortical bone that were almost 30% higher than those with a steel nail. The maximum stress in the nail (Figure 6) at the level of the fracture site did not vary significantly according to the material of the nail. The same was the case for the stress supported by the cortical bone in the distal region of the femur, where the stress difference when the distal screw was made of steel versus titanium was essentially nondiscernible.

## 4. Discussion

Frequently, the main indication for choosing one type of distal locking versus another lies in the degree of comminution and instability of the fracture, with preferential use in clinical practice of static locking when the fracture is considered relatively unstable. For this reason, the surgeon may also decide to delay the immediate weight-bearing.

To explain this effect at the mechanical level, the maximum overall mobility in the femoral head is not influenced by the type of distal locking. However, in the fracture site, the difference in mobility that we found depending on the type of locking was more noticeable (Table 3). We observed that the use of dynamic locking with steel nails increased the mobility at the fracture site by 11.46% (35 µm).

The stress experienced by the nail was not affected by the type of distal locking, as it depends fundamentally on the nail–bone contact surface. Table 3 shows how the difference in stresses in the nail depending on the type of locking did not reach 1%. Meanwhile, the stress experienced by the cortical bone in the distal region was affected at the level of the distal locking screws. Dynamic steel osteosynthesis withstood 27.16% more stress in the cortical bone at the level of the dynamic distal screw than the stress supported by the cortical bone around the static screws. This is because the static locking consisted of two screws, thus favoring the distribution of loads and halving the stress transmitted through the bone–nail and bone–screw interface.

Therefore, we can assume that distal locking fundamentally affects mobility at the fracture site, allowing for greater movement when dynamic locking is used, albeit at the expense of generating greater stress in the cortex around the distal screw than when two screws are used (static). However, it can be seen from the stress map (Figure 7) that the stress values were higher in the case of dynamic distal locking, and they were also concentrated in the cortical bone around the screw. However, with static locking, the stress values were spread throughout the supracondylar posterior region of the femur and not only around the screws.

There have been no studies to date assessing which type of distal locking is most beneficial for the treatment of fractures of the subtrochanteric region. In addition, there have been very few published studies to date that specifically compared the biomechanical and clinical effects of the intramedullary nail locking configurations [16,17,34,35]. In 1999, Brumback et al. [36] published the results of a study for which the main objective was to test the feasibility, safety, and efficacy of immediate loading after the treatment of diaphyseal femur fractures with intramedullary nail with static locking. These authors carried out a biomechanical study with 11 models that simulated fractures of the femoral shaft treated with nails with various types of distal locking, and they concluded that constructions involving a single screw (dynamic) exhibited relatively lower fatigue resistance (*p* < 0.05) than models with two screws (static). In a subsequent prospective observational study [37], they measured the consolidation rate in 100 femoral diaphyseal fractures treated with intramedullary nails with static locking, and they concluded that static locking in fractures of the femoral shaft does not negatively affect the fracture consolidation process and that it is not necessary to routinely perform nail dynamization. They concluded that statically locked nails are safe and better withstand fatigue after loading, allowing loading to be applied at a very early stage without causing further complications.

Other similar studies from the 1990s have raised the same doubts regarding the need to use one or two distal screws in intramedullary nails in femoral fractures [38,39]. Hajek et al. [40] carried out a study of the mechanical characteristics (torsional and compression forces) experienced by intramedullary nails treated with one screw or with two. They found no significant differences between the two constructions. The various studies published to date on the type of nail locking have concluded that static locking is safe and does not hinder consolidation, regardless of the type of fracture treated [36,41].

Many materials are used in orthopedics (polymers, metals, ceramics, and alloys, such as titanium). These materials have excellent physical, mechanical, and tribological properties. In addition, these materials must be nontoxic, biocompatible, and corrosion resistant. Titanium, stainless steel, and CoCrMo alloys are the most widely used biomaterials for orthopedic applications [42].

Regarding the type of material used, steel osteosynthesis is more rigid and may be safer in terms of material fatigue and failure of osteosynthesis (Table 1). Moreover, titanium nails provide a degree of elasticity that is closer to the bone, thus reducing the bridging of forces, which can facilitate the formation of a callus. In Table 1, it can be seen that Young’s modulus, i.e., the rigidity of steel, was 40% higher than that of titanium, which explains why titanium osteosynthesis allows for greater overall mobility at the fracture site (Table 4). In our study, the overall mobility (maximum proximal fragment displacement) obtained was 3.97 mm with the steel nail and 5.32 mm with the titanium nail, amounting to a difference of 34.4%. Similarly, the average mobility at the fracture site was 326 µm for the steel nail and 520 µm for the titanium nail, amounting to a mobility difference of 56.7%.

Gabarre et al. [16], in their study about diaphyseal fractures treated with an antegrade intramedullary nail, analyzed the effect of the nail material. For fractures of the most proximal region of the femoral shaft, they found overall mobility values of 1.35 mm for steel nails and 1.65 mm for titanium nails. Similarly, the mobility at the site exhibited the same trend, with maximum axial mobility values at the site of 66.13 µm with steel nails versus 100.13 µm with titanium nails. In another similar study conducted in the same year on fractures of the most distal region of the femoral shaft [17], they found the same trend according to the nail material. Therefore, regardless of the location of the fracture along the femoral shaft, the overall movements and micromovements at the fracture site exhibit the same trend found in our study with respect to the material used.

The presence of micromovements at the fracture site is known to be an important aspect of consolidation. However, it is also known that excessive mobility can lead to the destruction of an incipient bone callus, as this excess mobility prevents the neoformation of blood vessels and the formation of the precursor tissues of the bone callus [43]. Therefore, the displacement between the bone fragments at the fracture site must be sufficient to promote bone formation without exceeding the movement threshold that hinders or prevents the formation of callus. There is a paucity of studies on the bone consolidation process of mobility figures that establish the threshold between these opposite effects [43,44,45]. Yamaji et al. [46], in an experimental study with sheep, found that 0.7 mm micromovements during the early phase of consolidation stimulated the formation of callus. With this single reference, the maximum displacement found in our study of 0.54 mm with titanium nails suggests that the use of titanium nails is beneficial for the consolidation process.

Regarding the stresses found in the cortical bone at the level of the cephalic screw (Table 4), they were almost 30% higher when we used the titanium nail. The maximum stress in the nail, at the level of the fracture site, did not vary significantly according to the nail material. This was because the transmission of loads was fully equivalent, and although the overall rigidity of osteosynthesis varied, with titanium nails being more flexible, the relative rigidity between nail and bone was still very high due to the difference in values between Young’s modulus of bone and that of both metals.

The greater stress in the cortical bone with titanium nails, which we interpret as being derived from the lower rigidity of this material, may have a protective effect against the loss of bone mass during the fracture consolidation process, as it should allow greater transmission of loads through the bone surrounding the implant. This concept is referred to in the literature as “stress shielding” [47] or osteoporosis due to the transfer of loads through an implant that is always stiffer than the bone, i.e., if the nail or the prosthesis is stiffer than the bone, it acts on it as a shield, preventing it from receiving the necessary stress or overload and, therefore, results in loss of bone mass by resorption. As a result of bone loss, loosening of the implant occurs, which leads to the fracturing of bone, and a second surgery is necessary to replace the implant. For this reason, biodegradable materials are developed and utilized to eliminate the need for secondary surgery after implantation. Many metal-based (iron, zinc, magnesium) and polymer-based biodegradable materials have recently gained significant attention for orthopedic applications [48]. Among these materials, magnesium-based and Zn-based materials are the most suitable biomaterials for implant fabrication. Magnesium-based biomaterials have excessive release and rapid degradation compared with Zn alloys [49]. Therefore, Zn-based alloys have been utilized by integrating bioactive surfaces to treat challenging bone diseases and improve mechanical and degradation properties [50].

Recently, Sha et al. [51] carried out a study to assess the effect of nail rigidity on the fracture consolidation process. They concluded that titanium nails are more appropriate to achieve mineralization and formation of bone calluses in osteoporotic bones. They further demonstrated that nails with low stiffness reduce the effects of stress shielding and are strong enough to provide the stability needed to maintain fracture alignment.

In light of the results obtained, further research could explore new configurations of distal locking in titanium nails applied to femur models with different lengths and morphologies. This would contribute to a better understanding of biomechanical behavior and potential improvements in the surgical technique to be used in the treatment of subtrochanteric femur fractures.

Since in vivo animal experimentation has limited extrapolation to humans due to anatomical differences and loading conditions, and similarly, the results of in vitro experiments on cadaveric bone or synthetic bone models can hardly be applied to humans because of the differences between in vivo and in vitro behavior, finite element (FE) simulations of different osteosyntheses allow for parametric studies to assess the main critical factors involved in their biomechanical behavior. This provides a quick and easily repeatable testing method in various conditions that are difficult to achieve experimentally. Consequently, a better understanding of the problem can be obtained. It is essential to note, however, that the outcomes of these simulations must be compared with clinical findings, considering biological and physiological factors, which have a strong influence on the behavior of osteosyntheses.

## 5. Conclusions

In summary, our mechanical study showed that titanium nails allowed for greater micromobility at the fracture site, which could act as a stimulus for the formation of callus and consolidation of the fracture. The higher stresses detected in the cortical bone with the titanium nails may contribute to less bone atrophy during the consolidation process. In addition, no significant differences in stress were found on the material when compared with steel nails, which means that the risk of failure of the materials is similar. All this indicates that the use of titanium nails is advantageous compared with stainless steel nails under the conditions of our simulation.

In this mechanical study, we confirmed that the type of distal locking mainly affects mobility at the fracture site and stress in the cortical bone around the distal screws, without in any case exceeding values that may compromise the viability of the assembly or that may result in detrimental effects (in terms of mobility at the fracture site) for the consolidation process. Therefore, based on the presented results, we can conclude that static locking is safe and does not hinder consolidation for the types of fracture treated.

## Figures and Tables

**Table 1:** Mechanical properties of the materials [9].

Material	Young Modulus (MPa)	Poisson Ratio	Tensile Strength, Yield (MPa)	Tensile Strength, Ultimate (MPa)
**Cortical bone**	20,000	0.30		
**Cancellous bone**	959	0.12		
**Steel 316 LVM**	192,360	0.30	690	860
**TitaniumTi-6Al-4V**	113,760	0.34	785	896

**Table 2:** Biomechanical models.

	Type of Fracture	Fracture Location	Distal Locking	Nail Material
**Model 1**	Inverse	Subtrochanteric	Static	Steel
**Model 2**			Dynamic	
**Model 3**	Inverse	Subtrochanteric	Static	Titanium
**Model 4**			Dynamic	

**Table 3:** Biomechanical study data depending on the type of distal nail locking.

	Distal Locking		Dynamic/Static (%)
	Dynamic	Static	
**Maximum displacement in the femoral head (mm)**	3.97	3.84	3.36
**Mobility at the fracture site (µm)**	340	305	11.46
**Maximum stress in the nail at fracture site (MPa)**	106.21	105.96	0.24
**Maximum stress in the cortical bone at the level of the distal screws (MPa)**	20.01	15.74	27.16

**Table 4:** Biomechanical study data depending on the type of nail material.

	Type of Material		Titanium/Steel (%)
	Steel	Titanium	
**Maximum displacement in the femoral head (mm)**	3.97	5.32	34.00
**Mobility at the fracture site (µm)**	340	535	57.35
**Maximum stress in the nail at fracture site** **(MPa)**	106.21	100.91	−4.99
**Maximum stress in the cortical bone at the level of the distal screws (MPa)**	20.01	19.19	−0.41

## Data Availability

The datasets generated or analyzed during the study are available from the corresponding author on reasonable request.
